# Soil pH and plant diversity shape soil bacterial community structure in the active layer across the latitudinal gradients in continuous permafrost region of Northeastern China

**DOI:** 10.1038/s41598-018-24040-8

**Published:** 2018-04-04

**Authors:** Baihui Ren, Yuanman Hu, Baodong Chen, Ying Zhang, Jan Thiele, Rongjiu Shi, Miao Liu, Rencang Bu

**Affiliations:** 1 CAS Key Laboratory of Forest Ecology and Management, Institute of Applied Ecology, Chinese Academy of Sciences, Shenyang Liaoning, 110016 China; 2 University of Chinese Academy of Sciences, Beijing, 100049 China; 3 Institute of Landscape Ecology, University of Münster, Heisenbergstr. 2, 48149 Münster, Germany; 4 State Key Laboratory of Urban and Regional Ecology, Research Center for Eco-Environmental Sciences, Chinese Academy of Sciences, Beijing, 100085 China

## Abstract

In the permafrost region of northeastern China, vegetation and soil environment have showed response to permafrost degradation triggered by global warming, but the corresponding variation of the soil microbial communities remains poorly investigated. Here, a field investigation in the continuous permafrost region was conducted to collect 63 soil samples from 21 sites along a latitudinal gradient to assess the distribution pattern of microbial communities and their correlation with environmental factors. High-throughput Illumina sequencing revealed that bacterial communities were dominated by *Proteobacteria*, *Acidobacteria*, *Bacteroidetes* and *Actinobacteria*. Both microbial richness and phylogenetic diversity decreased initially and then increased as the latitude increased. UniFrac analysis of microbial communities detected significant differences among latitudes. Variation partitioning analysis and structural equation models revealed that environmental variables, including geographic factors, plant-community factors and soil physicochemical factors, all played non-negligible roles in affecting the microbial community structures directly or indirectly. Redundancy analysis and boosted regression tree analysis further highlighted the influences of soil pH and plant richness on microbial community compositions and diversity patterns. Taken together, these results suggest that the distribution pattern of soil microbial communities shows distinct changes along the latitudinal gradients in northeastern China and is predominantly mediated by soil pH and plant diversity.

## Introduction

Permafrost, defined as soil that has remained frozen at least 2 consecutive years, covers approximately 25% of the land area on Earth and is estimated to contain around 50% of the global soil organic carbon^1^. However, the warming climate has accelerated the widespread and rapid degradation of permafrost during recent years, posing a growing risk of losing this soil carbon because of the acceleration in decomposition of soil organic matter^2^. Although permafrost is thought to be an inhospitable environment, it in fact hosts numerous and various microorganisms, which assume vital part in controlling biogeochemical functioning, such as nutrient turnover and biomass production^3^. The microbial ecology of permafrost has become a focus of attention owing to concerns about the impact of the microorganisms on decomposition and mineralization processes in permafrost soil carbon stocks, particularly the potential to increase emissions of greenhouse gases, which as a consequence, would be expected to further exacerbate climate change^4–7^. The active layer, which is the surface of permafrost and is most sensitive to permafrost thaw, has gained much scientific attention due to the various biogeochemical, hydrological and ecological activities that dynamically occur in this layer^8^. Thus, it is especially critical to assess the variables that affect the structure and distribution of microbial communities in the active layer of permafrost to predict the potential consequences of climate change.

Studies in the Arctic and on the Qinghai-Tibet Plateau have shown that the distribution pattern of microbial communities in permafrost soils is linked to the corresponding environment^9–13^. As the fundamental energy sources and component elements for microorganisms, nutrient availability altered microbial community composition in Arctic tundra soils through determining the metabolism of soil microbes, especially of carbon and nitrogen cycling bacteria^9^. Soil moisture was the main factor underlying the variations in the microbial community structures of permafrost soils on the Tibetan Plateau by controlling the dominant vegetation and the soil redox potential, which were in terms of both the carbon utilization and turnover rates for microbial communities^11,14^. Soil pH has also been showed to be a primary determinant of microbial community composition and diversity in permafrost-affected soils^10,15,16^. Besides, plant communities may regulate microbial communities via the combined effects of nutrient supply and modification of the physical environment in the permafrost ecosystems^17–19^. Permafrost degradation triggered by climate warming has resulted in substantial changes in the soil environment as well as vegetation characteristics^20–22^. All these observations suggested a close association between the microbial community and environmental changes in response to permafrost degradation.

Located on the southeastern edges of the Eurasian cryolithozone, the permafrost in northeastern China, known as the main high-latitude cold area and the second largest expanse of permafrost in China, is thermally unstable and ecologically sensitive to climatic change^23^. There exist an obvious distribution pattern of latitudinal permafrost^24^. With latitude decreases, the mean annual ground surface temperature and the thickness of the active layer increases. At the same time, permafrost changes from continuous, discontinuous to sporadic. Because of climatic warming and human activities, the southern limit of permafrost in this region has retreated and will still retreats northward^23,25^. This degradation of the permafrost environment has subsequently influenced the composition of plant communities, which has important implications for ecosystem functioning and carbon storage^26,27^. However, the distribution pattern of microbial communities in this unique permafrost soil remains largely unexplored and the variation in the structures of microbial communities and their relationships to the environmental properties are not clear.

In the present study, we examined the latitudinal distribution of bacterial communities in the top active layer soils with the high-throughput Illumina sequencing method and analyzed their correlations with environmental characteristics in continuous permafrost region of northeastern China. We aimed to address the following questions: (1) How do microbial communities distribute in the latitudinal permafrost? (2) What are the key factors that determine microbial community structures in the active layer of permafrost soils?

## Results

### Partitioning of permafrost soil microbial community structures along a latitudinal gradient

**Figure 1:**
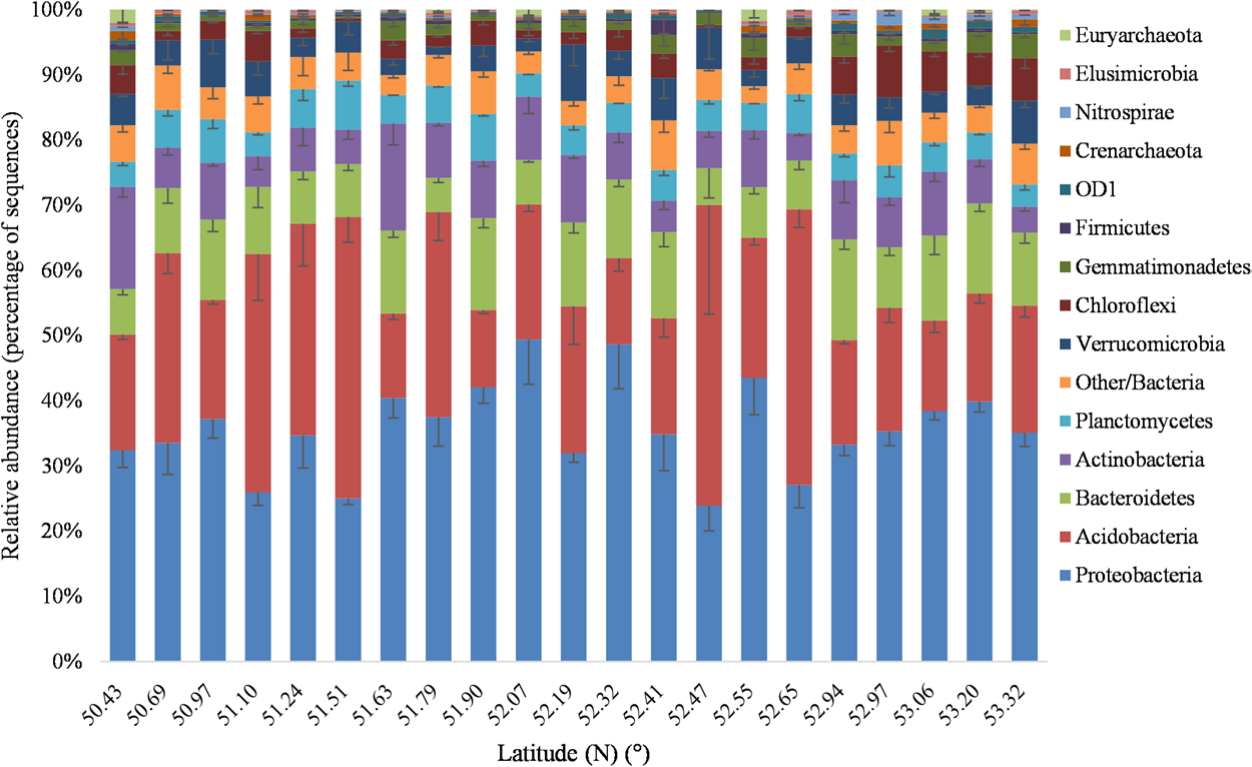
Relative abundance of dominant phyla of bacteria and archaea in permafrost soils along a latitudinal gradient.

Across all the examined samples, the high-throughput Illumina sequencing yielded a total of 646,773 high-quality 16 S rRNA gene sequences. The sequences were binned into 146,881 OTUs at 97% sequence identity, which belonged to 58 phyla. The microbial community composition was profiled according to their relative abundance at the taxonomic levels of phylum (Fig. 1). And the 3 replicates agreed well in terms of community structure for each site (see Supplementary Fig. S1). Among those taxa examined, *Proteobacteria* (34.86%), *Acidobacteria* (23.33%), *Bacteroidetes* (10.04%) and *Actinobacteria* (7.86%) were the most dominant phyla of the bacterial community in all samples. The main four bacterial phyla accounted for >67.81% of the bacterial sequences from each of the soils. In addition, each sample also contained expansive number of sequences that could not be classified, even at the phylum level.

Although most of the microbial groups were present in all samples, the relative abundances of the dominant bacterial taxa varied along latitudinal gradients (see Supplementary Fig. S2). In general, the abundances of *Proteobacteria* and *Bacteroidetes* showed an increasing trends, whereas the trends of the abundances for *Acidobacteria* and *Actinobacteria* decreased along the increasing latitudes.

The non-parametric PERMAVONA test was conducted to test the significance of differences in community composition along the latitudinal gradient. The composition of bacterial communities varied significantly among different sample latitudes (PERMANOVA, *P* < 0.01).

**Figure 2:**
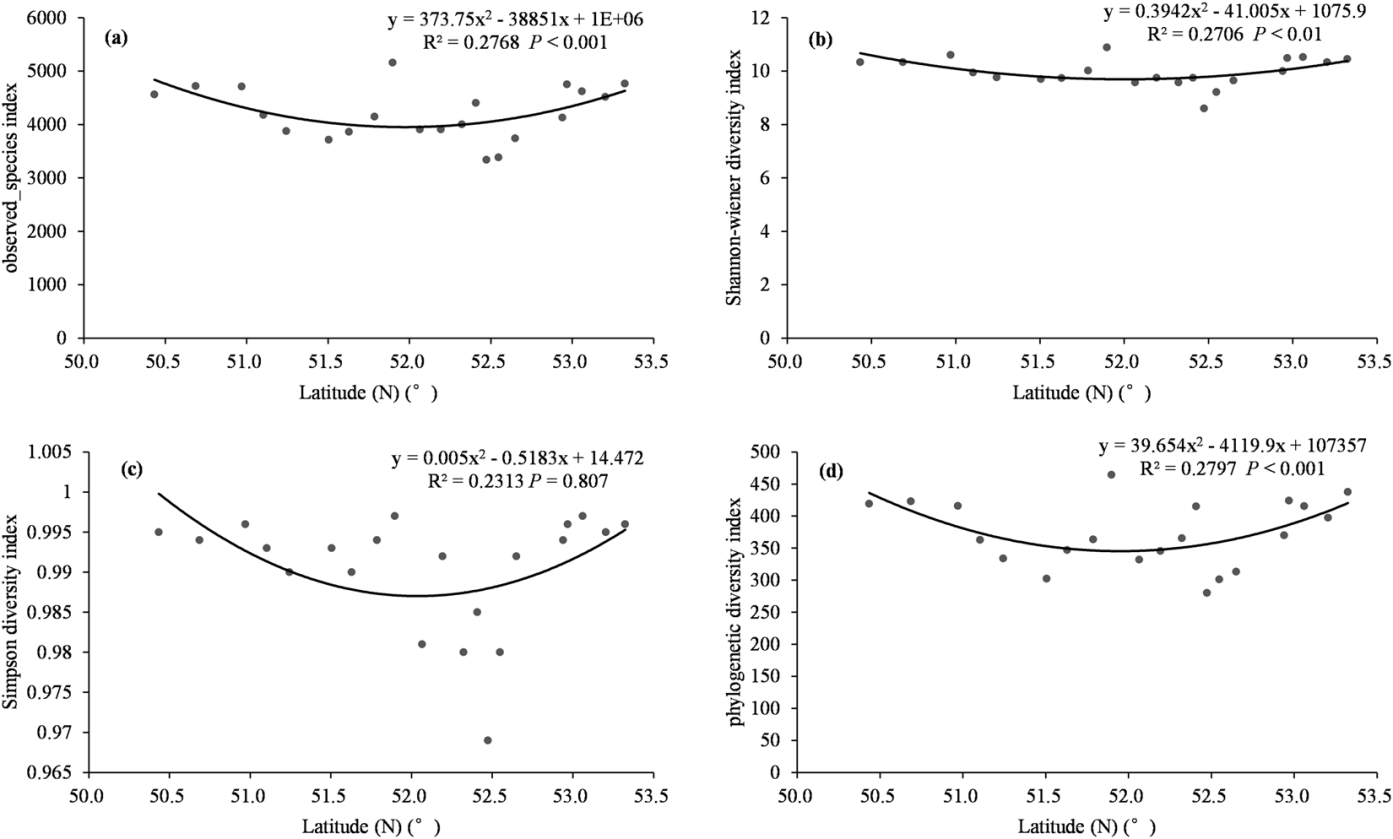
Diversity patterns of permafrost soil microbes along latitudinal gradient as characterized by (**a**) observed species index, (**b**) Shannon diversity index, (**c**) Simpson diversity index and (**d**) phylogenetic diversity index.

To explore the latitudinal diversity patterns of microbial communities, we fitted curves over the latitude gradient for observed_species index, Shannon diversity index, Simpson diversity index and phylogenetic diversity index (Fig. 2). The values of these four indices all decreased and then increased again with increasing latitude. Hence, the diversity of microbial communities was lowest at intermediate latitude.

### Correlations between environmental variables and microbial community structures

**Figure 3:**
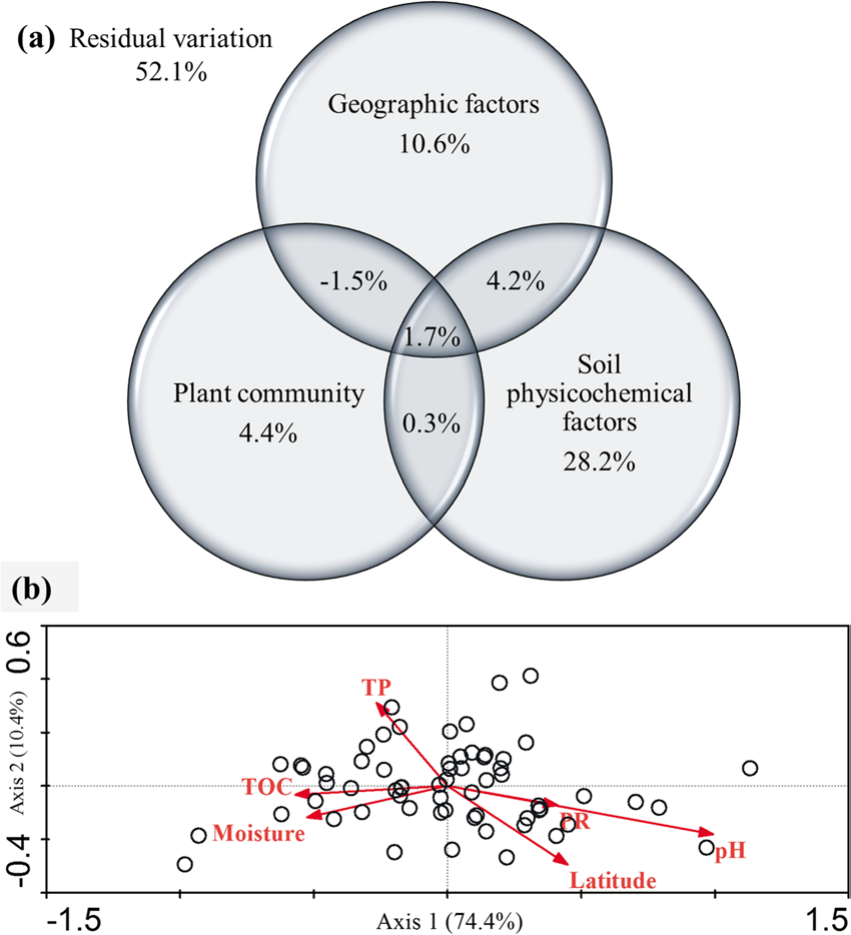
Variance partitioning (**a**) and biplots (**b**) based on redundancy analysis of the microbial community compositions and environmental factors. (PR, plant richness; TP, soil total phosphorus; TOC, soil total organic carbon).

A variance partitioning analysis was carried out to assess the relative contributions of geographic factors, plant community factors and soil physicochemical factors to microbial community composition (Fig. 3a). The combination of these variables explained 48.9% of the observed variation in soil microbial community composition. Soil physicochemical factors explained the largest fraction of the variation (34.4%), with a pure effect of 28.2%. Geographic factors and plant community factors explained 15.0% and 4.9% of the variation, respectively.

We also used redundancy analysis (RDA) to simultaneously measure the relative importance of each single environmental variable for microbial community composition (Fig. 3b). The first two axes accounted for 74.4% and 10.4% of the variance for microbial community, respectively. Of all the soil environmental factors examined, soil pH (27.9%), soil total organic carbon (TOC, 4.7%), soil total phosphorus content (TP, 4.7%), Moisture (4.7%), plant richness (PR, 4.7%) and Latitude (2.3%) were the most significant parameters underlying the variations in the microbial community composition.

**Figure 4:**
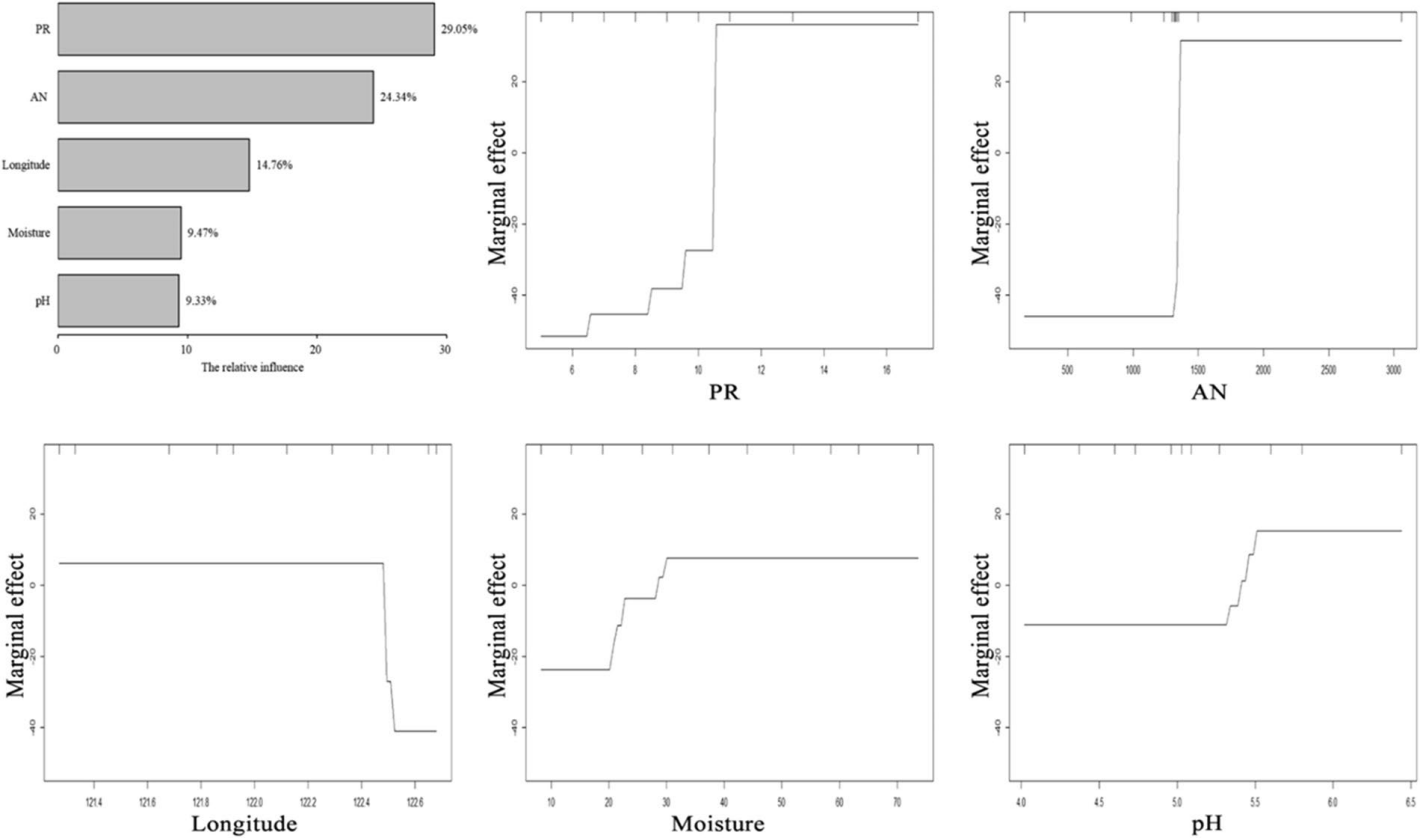
Relative influence on soil microbial richness and dependency plot for the first five most important predictor variables in a boosted regression tree analysis. Each dependency plot represents the marginal effect of a predictor variable on soil microbial richness. Marginal effects were constrained in the model to be monotonic. (PR, plant richness; AN, soil available nitrogen).

The influences of the relative importance as well as the marginal effects of individual environmental factors on microbial diversity were performed by boosted regression tree (BRT) analysis (Fig. 4). The first five most important factors influencing microbial richness were PR, soil available nitrogen (AN), Longitude, Moisture and soil pH, and the relative influence of those variables were 29.05%, 24.34%, 14.76%, 9.47% and 9.33%, respectively. Generally, an increase in PR, AN, Moisture and soil pH resulted in higher microbial richness. The sites with higher Longitude exhibited a decrease in microbial richness.

**Figure 5:**
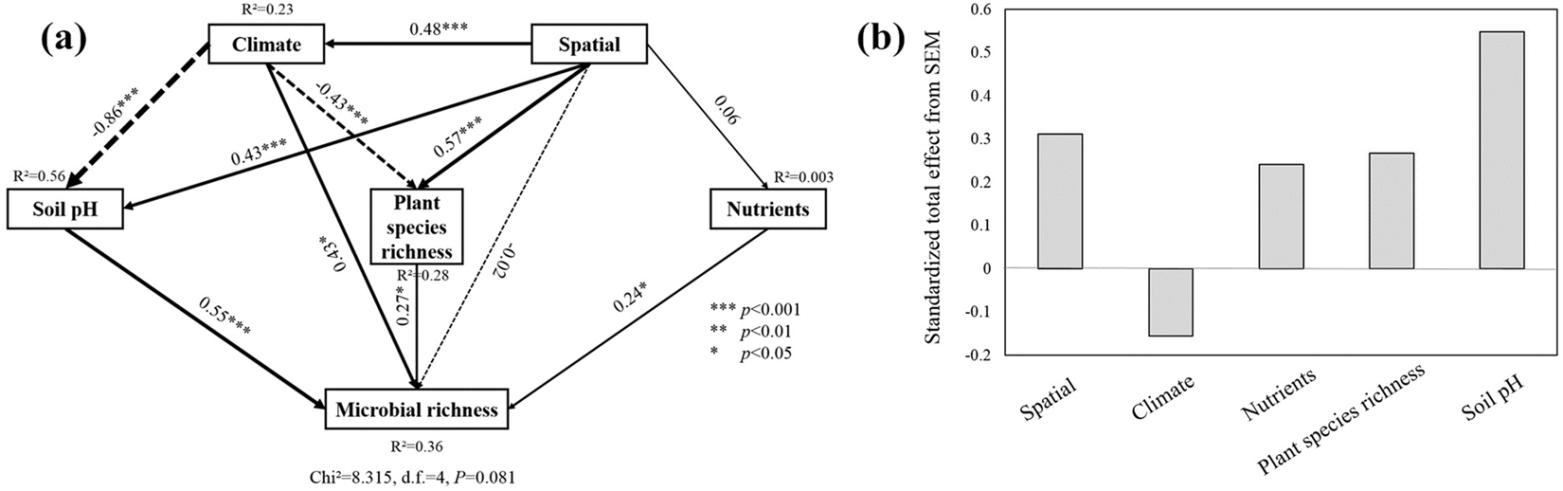
Structural equation model showing the direct and indirect effects of spatial factors, climate, soil pH, nutrients and plant species richness on soil microbial richness (**a**), and their standardized total effects (direct plus indirect effects) derived from the structural equation model of microbial richness (**b**). Numbers adjacent to arrows are path coefficients, and width of the arrows is proportional to the strength of path coefficients. Continuous and dashed arrows indicate positive and negative relationships, respectively. Significance level: *P < 0.05; **P < 0.01; ***P < 0.001.

Considering the collinearity of factors in our study (Table S1), we used structural equation models (SEM) to explore how soil microbial diversity was associated with climate factor, spatial factor, soil pH, soil nutrient content and plant diversity and to assess the direct and indirect effects of explanatory variables on microbial richness (Fig. 5). The fitted models explained 36% of the variance in microbial richness (Fig. 5a). Soil pH, climate, plant species richness and nutrients were identified as significant drivers that directly structure the microbial richness. Standardized total effects derived from the SEM revealed that microbial richness was mainly driven by soil pH, followed by spatial, plant species richness, nutrients and climate (Fig. 5b).

## Discussion

Our high-throughput Illumina sequencing results, combined with our landscape-level sampling design demonstrated conclusively that soil microbial community exhibited changes both in composition and diversity along the latitudinal gradients.

*Proteobacteria*, *Acidobacteria*, *Bacteroidetes* and *Actinobacteria* (Fig. 1) were the main phyla found in the active layer soils in continuous permafrost region of northeastern China. Similar community composition were also reported from permafrost-affected soils by some other studies in northeastern China, such as on the Sanjiang Plain^28^, in Mo-he^29^, and along the China-Russia Crude Oil Pipeline^30^. These results were also mostly in agreement with previous studies that were conducted in Tibetan^11,14^ and Arctic^10,31^, indicating that these groups of bacteria were well adjusted to the extreme conditions of permafrost habitats. The overwhelming first principal component of the redundancy analysis (Fig. 3b) generally indicated that there was a very high degree of correlation between the many phyla, or between at least two phyla while the others showed a much smaller dispersion, which was in accordance with Fig. 1. Although most bacterial groups were relatively stable across latitudes, bacterial community compositions differed significantly among latitudes (PERMAVONA*, P* < 0.01) and the dominant phyla exhibited changes in their relative abundance (Fig. 1 and Supplementary Fig. S2). For example, we found that the relative abundance of *Proteobacteria* increased, whereas the trends of the abundances for *Acidobacteria* decreased as latitudes increased (see Supplementary Fig. S2). This observation might correspond to soil pH and soil total organic carbon, which were both significantly positively correlated with latitude (see Supplementary Table S1). Previous studies demonstrated that *Acidobacteria* favored acidic conditions and were negatively correlated with pH, whereas *Proteobacteria* dominated in higher pH soils^10,15,32^. Further, it has been shown that more labile pool of bioavailable organic carbon in the near-neutral soils support copiotrophic bacteria like *Betaproteobacteria*, whereas the less bioavailable organic carbon in acidic soils favored bacteria like *Acidobacteria*^10,18,31,33^.

We observed that both microbial richness and phylogenetic diversity decreased initially and then increased as the latitude increased (Fig. 2). Several researches reporting a distinct distribution pattern of microbes have emphasized the great importance of environmental variables^34–36^, which made decisive contributions to structuring microbial community compositions^37^. In our study, correlation analyses revealed that environmental factors exhibited an obvious distribution trend along the latitudinal gradient (see Supplementary Table S1). Apparent diversity pattern of microbial community highlighted the importance of these environmental factors in regulating microbial distribution. Microbial communities are made up of species with different adaptive capacities to their habitats. Differences in response to environmental factors in these species may bring about differences in patterns of community distribution, which may change along the environmental gradients. Degradation of permafrost could significantly change edaphic conditions and vegetation composition^38^, which could lead to a disparity in ecological niche for microbial species and further generate changes of species composition and diversity of microbial community. In addition, the Greater Hinggan Mountains in permafrost zone of northeastern China generate mountain effect^25^ and thus may affect the diversity pattern of soil microbial communities.

In our investigation, environmental variables including geographic factors, plant community factors and soil physicochemical factors all played certain roles in determining microbial community structures in the active layer of permafrost soils at the regional scale of northeastern China (Figs 3a and 5). Of all the factors we examined across the range of statistical analyses, soil pH and plant richness gained more attention for their considerable influences on both microbial community compositions (Fig. 3b) and diversity patterns (Figs 4 and 5).

The soil pH is known to have a strong effect on the structure of soil microbial communities at all scales^15,17,35,39^ and also in the permafrost ecosystems of Arctic and Alpine regions^10,31,40,41^. As the most imperative characteristic of soil, pH influences soil microorganisms by modifying enzymes activity as well as by controlling the accessibility of nutrient and moisture supplements through deciding the ionization balance in soil^32^. For example, pH decided the community composition and biogeography of ammonia oxidizers by influencing ionization equilibrium of nitrate and ammonia in soils^42^. Furthermore, studies found that soil pH was the main factor that influenced community composition and diversity, which may be connected to the relationship between soil pH and the relative abundances of dominant bacterial groups, such as *Acidobacteri*^10,15^. Soil pH-driven microbial distribution patterns were observed across biomes or along the altitudinal gradient^35,36^, and our research corroborates that it could have great impact on microorganism across the latitudinal gradients.

Aboveground plant communities, which are tightly associated with belowground microbial communities to form a systematic entity to maintain ecosystem functions, strongly affect soil microbial community structure through the consolidated impacts of nutrient accessibility and the physicochemical state of the soil^17–19,43^. Higher plant richness is normally connected with higher plant productivity because of niche complementarity and more resource supply^44^, resulting in increases in the nutrient pools that are available to microorganisms, and thus favor higher microbial richness^45,46^. In our study it may have been the case that plant richness was a key factor, which related to soil nutrient availability, responsible for differences in soil microbial community diversity and composition along the latitudinal gradient. This was most likely in light of the fact that more nutrient availability in higher latitude soils, derived from the denser vegetation litter and/or root exudates, could boost microbial community diversity, such as specific rhizosphere bacteria and symbiotic bacteria^40,47^. Previous studies had shown that permafrost degradation would potentially change plant communities and the corresponding soil physicochemical environment^48–51^. Therefore, the latitudinal gradient in our research structured the community of plant, and variation in plants, which were concomitant with soil properties, might lead to distinct microbial communities.

In addition, soil moisture and nutrients of TOC, TP and AN also played non-negligible roles in affecting the microbial community structure (Figs 3b and 4) via determining the metabolism of soil microbes^52–54^. The freezing and thawing process in the active layer of permafrost may have an impact on soil microbial communities, not just by straightforwardly influencing the metabolic activity and reproduction of soil microorganisms, but, additionally, by changing soil physical properties like temperate, moisture, and rock weathering that firmly related with soil mechanical composition^1^. The plant exudates in the rhizosphere at top active layer of permafrost could also be important to shape microbial structure by influencing soil properties like TOC, TP and AN.

Climatic factor played an important role and had a significant direct influence on soil microbial communities. Meanwhile, it also indirectly affected soil microbial communities via strong correlations with soil pH and plant richness (Fig. 5a). Permafrost in Northeastern China is mainly the result of latitudinal climate zonation and is susceptible to the impact of changes in climate due to its location in the southeastern margin of the Eurasian cryolithozone. A study^23^ predicted that thawing and degradation of permafrost would occur with a reduction of 49.8% in the permafrost area according to the observed trend of climate warming and the regional climate change scenarios in the next 100 years. As the permafrost thaws and the organic matter turns out to be more available to microorganisms, microbial activity and diversity also increase^6,55^. This could bring about a huge positive feedback to the greenhouse effect and may adversely affect ecological environments in cold regions.

What’s more, since environmental variables shape microbial community structure (Figs 3a and 5), such distinct distribution pattern of microbial community in our study would be the coefficient outcome of all the ecological processes. At regional scale, harsh climatic conditions make the latitudinal permafrost in northeastern China a particular biogeographic region and a unique as well as fragile ecosystem. Spatial geographical distance and climatic factors plays key roles in regulating ecosystem functioning and affecting aboveground net primary production of the plant community, along with soil physicochemical factors, which in turn affects the quantity and quality of plant residue input to below-ground microbial communities^56,57^.

In summary, we conducted a comprehensive study to investigate the distribution pattern of microbial communities and the corresponding driving factors along the latitudinal gradients in the active layer of permafrost soils of northeastern China. Our results implied that latitudinal variation could have important and dramatic effects on microbial community compositions and diversity patterns. We also found that soil pH and plant diversity predominantly explained the variability of soil microbial community structures. Our study strengthens our knowledge about the diversity and ecology of microorganism in permafrost ecosystem of northeastern China. More efforts should be taken to provide further insights into the factors that drive microbial community structures in this unique permafrost area, which in the end would be beneficial for ecologically sound conservation of the high-latitude cold ecosystems under current climate change.

## Methods

### Site description, field investigation and sampling

The study area was located in the continuous permafrost zone of northeastern China and comprised the forest ecosystems, with a relatively abundant species composition. The vegetation mainly consisted of cold-resistant and hygrophilous species, including two tree species, *Larix gmelinii* and *Betula platyphylla*, three important shrubs, *Betula fruticosa*, *Ledum palustre* var*. angustum* and *Vaccinium uliginosum* and a dominant herb, *Carex subpediformis*^26^. The mean annual air temperature and precipitation ranged from −4 to −1 °C and from 350 to 550 mm, respectively.

**Figure 6:**
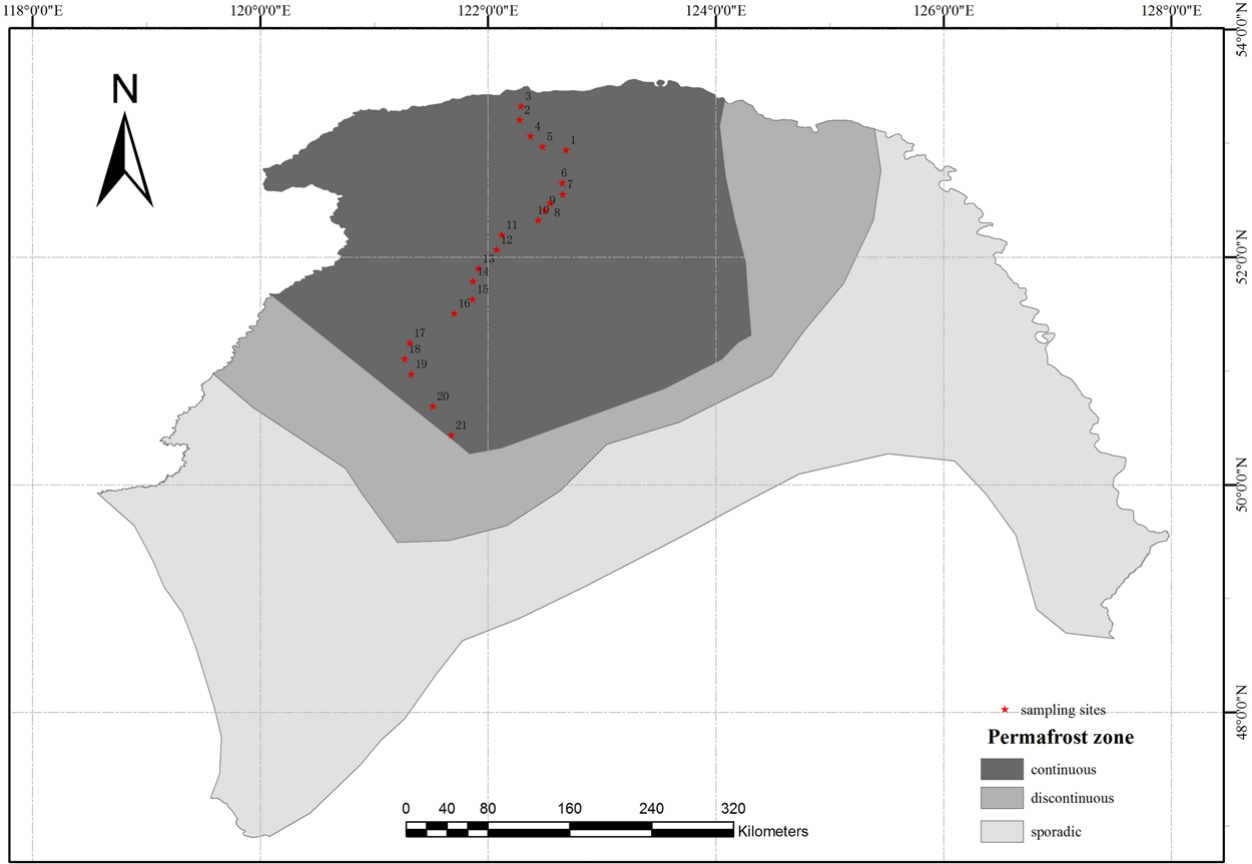
Study area and location of the 21 sampling sites in the Great Hing’an Mountain area of northeastern China. The map in this figure was generated by ArcGIS 10.2 (ESRI, Redlands, CA, USA, http://resources.arcgis.com/en/home/). Permafrost distribution in this map was produced using the original data source of Jin, *et al*.^25^.

Field investigations were conducted during the growing season in August of 2015. Soil samples were collected from 21 sites along a latitudinal gradient (Fig. 6). At each site, spatial geographic coordinates (longitude and latitude) and elevation were recorded using a GPS device (eTrex Venture, Garmin, USA). Three randomly chosen 2 × 2 m^2^ plots within an area of 20 × 20 m^2^ were selected at each site and, in each plot, five soil cores were collected (20 cm deep and 5 cm in diameter) and combined to form a single sample, resulting in 63 soil samples (3 plots × 21 sites). Soil temperature, moisture and salinity were synchronously detected by an *in-situ* soil testing device (TZS-PHW-4, China). To obtain plant composition data, we identified all the understory plant species and counted the total number of species within each plot. After harvesting all above-ground plant parts, we measured plant biomass after oven-drying. All samples were cooled with ice packs and transported to the laboratory immediately. After thorough homogenization, the composite soils were sieved (2 mm) to remove fine roots and large organic debris and divided into two portions. One portion was stored at 4 °C for characterization of soil physical and chemical properties, and the other portion was stored at −80 °C for DNA extraction.

### Soil physicochemical analyses and climate data acquisition

Soil pH was determined in a 1:2.5 (w/v) aqueous solution. Soil total nitrogen content (TN) and total carbon content (TC) were measured with an element analyzer (EL Ш, Elementar, Germany). Then, soil carbon: nitrogen (C/N) stoichiometry was calculated based on TC and TN. Soil total phosphorus content (TP) was determined using the Mo-Sb anti-spectrophotometry method after digestion with concentrated HClO_4_–H_2_SO_4_^58^. Soil total organic carbon (TOC) was measured using a K_2_Cr_2_O_7_ oxidation method according to Walkley^59^. Soil available nitrogen (AN) was extracted with 2 M KCl (soil to water ratio of 1:5) and measured using a continuous flow analyzer (SAN++, Skakar, Breda, Holland). Soil available phosphorus (AP) was measured using the method^60^ after extracted with 0.5 M NaHCO_3_. Soil texture was determined by a particle size analyzer (Malvern Instruments, Malvern, UK) according to the universal criteria of soil particle size (clay <0.002 mm, silt 0.02–0.002 mm, sand >0.02 mm). The climatic data were compiled for 1980–2010 from 78 weather stations in northeastern China using the spatial interpolation method as described in the study of Ma, *et al*.^61^. We used ArcGIS 10.2 to interpolate climatic data into raster-based spatial distribution data and, then, we extracted all data values of mean annual temperature (MAT) and mean annual precipitation (MAP) for each sampling site. Supplementary Fig. S3 lists the ranges and explanations of all the environmental variables.

### DNA extraction, PCR amplification, and Illumina-based sequencing

The total DNA of each soil sample was extracted by PowerSoil DNA Isolation Kit (MOBIO, USA) according to the manufacturer’s instructions. After qualitative detection with agarose gel electrophoresis and determination by Nanodrop2000 (Thermo, USA), the V4 hypervariable region of 16 S rRNA gene was amplified using the primers of 515 F (5′-GTGCCAGCMGCCGCGGTAA-3′) and 909 R (5′-CCCCGYCAATTCMTTTRA GT-3′) with a unique barcode. The process of PCR amplification was performed as described in a previous study^62^. The PCR products were purified with the AxyPrepDNA Gel Extraction Kit (AXYGEN, California, USA). The resultant PCR products were combined at equimolar concentrations before being sequenced on an Illumina Miseq platform at Chengdu Institute of Biology, Chinese Academy of Sciences.

### Sequence analysis

The obtained raw sequences ranged from 12,035 to 48,670 per sample were processed following the Quantitative Insights Into Microbial Ecology (QIIME) pipeline (QIIME v.1.8.0; http://www.qiime.org). Prior to clustering, reads with quality score lower than 20, improper primers, or ambiguous bases were removed, and chimeras were identified and eliminated. The resultant high quality sequences that shared at the level of 97% similarity were clustered into operational taxonomic units (OTUs). De novo OTU clustering was done with the method of uclust^63^. The PyNAST method was used for sequences alignment. Taxonomy assignments were based on the 13_8 Greengenes database and their respective taxonomy files^64^. The community compositions was then described by the relative abundance of sequences that were assigned to each taxon. Taxa filtering script provided by QIIME was used to separate the OTU tables of individual microbial taxa, according to which the diversity of specific taxa was analyzed. To eliminate the bias due to sampling size, a randomly selected subset of 10,603 sequences in each sample were used for the downstream analysis. The sequencing depth was sufficient to cover all the samples reflected by the rarefaction curve of Shannon diversity (see Supplementary Fig. S4). The observed_species index (microbial richness), Shannon diversity index, Simpson diversity index and phylogenetic diversity index^65^ were calculated to compare the alpha diversity. The unweighted UniFrac distance^66^ was used as beta-diversity metric.

The DNA sequences in this study have been deposited in the National Center for Biotechnology Information (NCBI) Sequence Read Archive (SRA) database under accession number SRP129906 (one sample was excluded because of low quality).

### Statistical analyses

A permutation multivariate analysis of variance (PERMANOVA) based on the unweighted UniFrac matrix was performed to test the significance of the divergence in microbial community compositions between the 21 latitude groups. We implemented a variation partitioning analysis to assess the relative importance of each environmental driver (geographic factors: Latitude, Longitude, Elevation, MAT and MAP; plant community factors: PR and PB; soil physicochemical factors: Temperature, Moisture, Salinity, TC, TN, TP, C/N, TOC, AN, AP, pH, Clay, Silt and Sand). All these analyses were carried out with the “vegan” package in R^67^.

The effects of environmental variables on microbial community structures were analyzed by constrained ordination redundancy analysis (RDA) in CANOCO 4.5 software (Microcomputer Power, Ithaca, NY, USA). A Monte Carlo test (999 permutations) based on the RDA was used to assess the effects of each variable. Factors that had no significant effects on community structure were excluded from RDA plots.

Spearman’s correlation analysis was performed to examine the relationships between microbial richness and environmental variables using the software SPSS 20.0 (IBM Co., Armonk, NY, USA).

Boosted regression tree (BRT) analysis was used to explore the relative importance as well as the marginal effects of individual environmental factors influencing microbial richness with the “gbm” package in R. Joining the benefits of regression trees and boosting, BRT analysis is a machine-learning approach in view of the classification and regression trees. It has strength in tending to nonlinear relationships^68^, and this complex nonlinear relationship can be reflected by the marginal effect and the relative impact of each independent variable on response variables created during BRT examination. The marginal effect of an individual predictor variable is ascertained based on the presumption that other independent variables are steady, and this impact will be appeared in a dependency plot. In this study, microbial richness was selected as the response variable and environmental variables including geographic factors, plant community factors and soil physicochemical factors were set as predictor variables. Parameters including Gaussian error distribution, a learning rate of 0.01, a bag fraction of 0.75, and fivefold cross-validation were set in BRT investigation. We only present the initial five most imperative predictor variables affecting the response variable in the outcomes.

Considering the collinearity of factors across spatial scales, we used structural equation models (SEM) to estimate the direct and indirect effects of climate factor, spatial factor, soil pH, soil nutrient content and plant diversity on the microbial richness according to hypothesized causal relationships and the knowledge of previous study by Wang, *et al*.^69^, using the Amos 21.0 software (Smallwaters Corporation, Chicago, IL, USA). The explanatory variable of climate was a synthetic variable derived from the first axis of a principal component analysis (PCA) of MAT and MAP, and the variable of nutrients was derived analogously from TC, TN, TP, TOC, AN and AP. Geographic distance was obtained from Principal Components of Neighbor Matrices (PCNM), and used as the explanatory variable of spatial factor. PCA and PCNM were performed using of “psych” and “vegan” package in R. Sufficient model fits of SEM were determined by a non-significant χ^2^ test (P > 0.05), low Akaike value (AIC), high goodness fit index (GFI) (>0.90), and low root mean square error of approximation (RMSEA) (<0.05)^70^. Standardized total effects of climate, spatial, soil pH, nutrients and plant species richness on the microbial richness were also calculated for SEM.

## Electronic supplementary material

Supplementary Information
