# Supplementary material for: Soil pH and plant diversity shape soil bacterial community structure in the active layer across the latitudinal gradients in continuous permafrost region of Northeastern China
Source: Sci Rep. 2018 Apr 4;8:5619. doi: 10.1038/s41598-018-24040-8 (PMC5884794; doi:10.1038/s41598-018-24040-8)
Supplement: Supplementary file 1 — Supplementary Information [file 41598_2018_24040_MOESM1_ESM.pdf]

Supplementary Information

Soil pH and plant diversity shape soil bacterial community structure in the active layer across the latitudinal gradients in continuous permafrost region of Northeastern China

Baihui Ren[1, 2, 3], Yuanman Hu[1], Baodong Chen[2, 4], Ying Zhang[1], Jan Thiele[3], Rongjiu Shi[1], Miao Liu[1], Rencang Bu[1, *]

1. CAS Key Laboratory of Forest Ecology and Management, Institute of Applied Ecology, Chinese Academy of Sciences, Shenyang Liaoning 110016, China
2. University of Chinese Academy of Sciences, Beijing 100049, China
3. Institute of Landscape Ecology, University of Münster, Heisenbergstr. 2, 48149 Münster, Germany
4. State Key Laboratory of Urban and Regional Ecology, Research Center for Eco-Environmental Sciences, Chinese Academy of Sciences, Beijing 100085, China

* Corresponding author.

E-mail: rencangbu@163.com; burc@iae.ac.cn;

Tel: +86 024 8397 0350;

Fax: +86 024 8397 0350;

Postal address: No. 72, Wenhua road, Shenhe district, Shenyang, Liaoning 110016, China

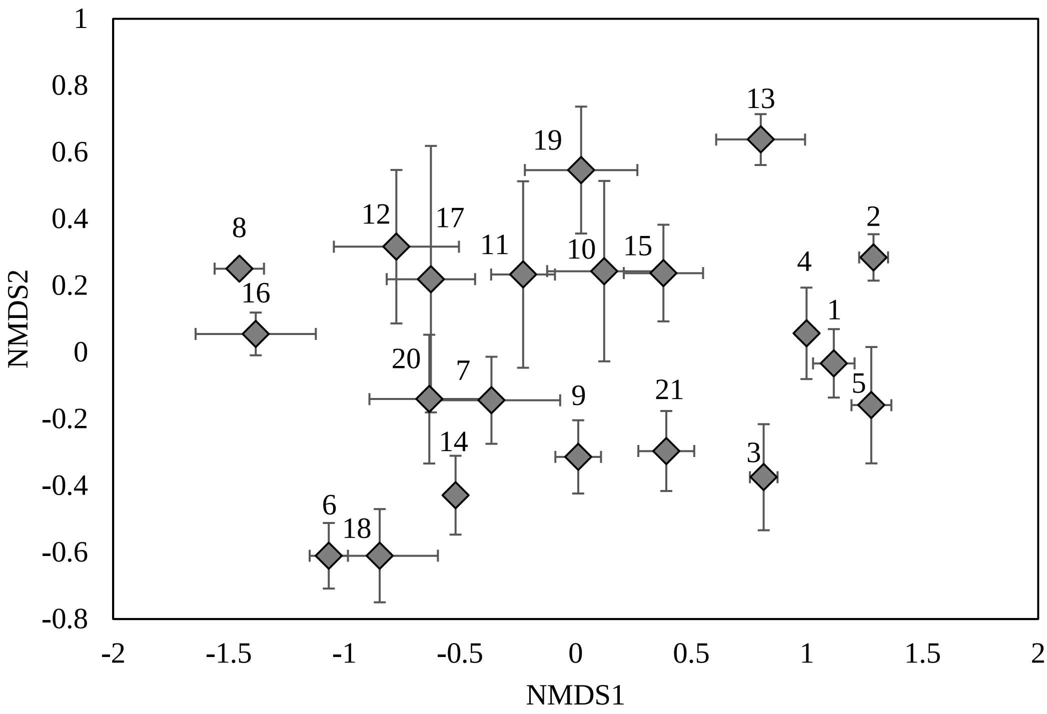

Supplementary Figure S1. Nonmetric multidimensional scaling (NMDS) averaged by site (Stress=0.07), error bars represent standard error of three replicates within each site. Analysis was carried out based on the unweighted UniFrac distances matrix using the nmds.py script in QIIME.

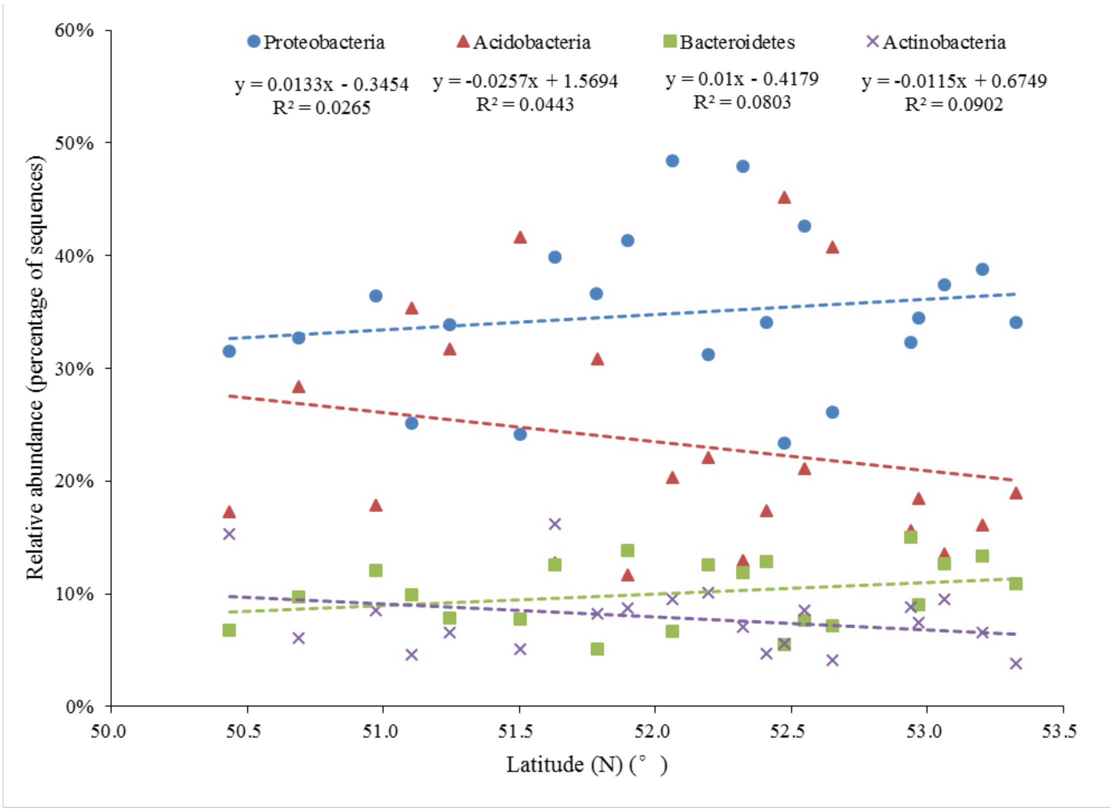

Supplementary Figure S2. The relationships between relative abundances of dominant bacterial groups and latitude. Linear regressions were used to test the correlation between the taxa's relative abundances and latitude.

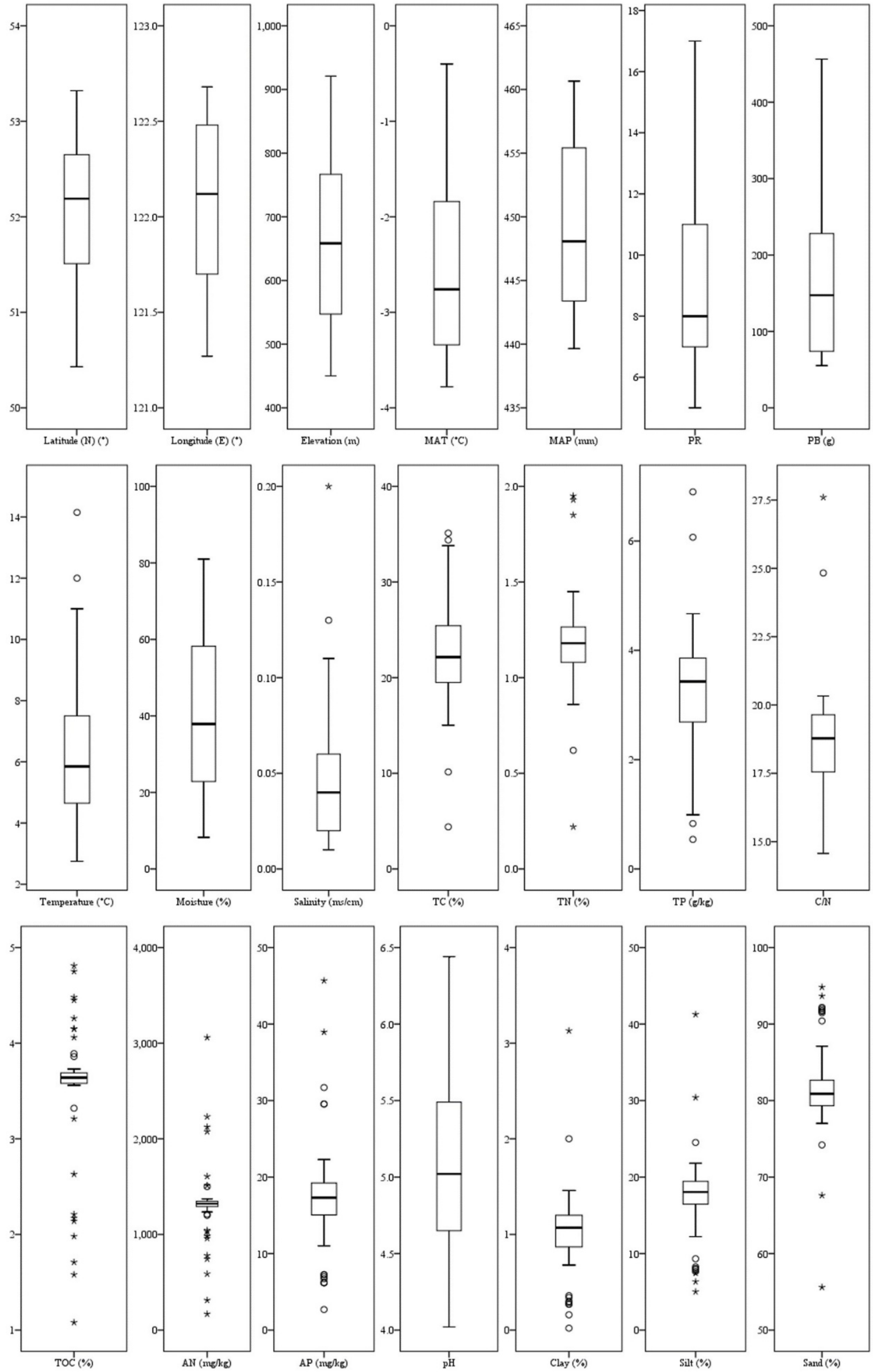

Supplementary Figure S3. Boxplots of environmental variables for the sampling

samples. (MAT, mean annual temperature; MAP, mean annual precipitation; PR, plant richness; PB, plant biomass; TC, soil total carbon; TN, soil total nitrogen; TP, soil total phosphorus; C/N, soil C:N stoichiometry; TOC, soil total organic carbon; AN, soil available nitrogen; AP, soil available phosphorus; Clay, soil particle size <2μm; Silt, soil particle size 2~20μm; Sand, soil particle size >20μm.)

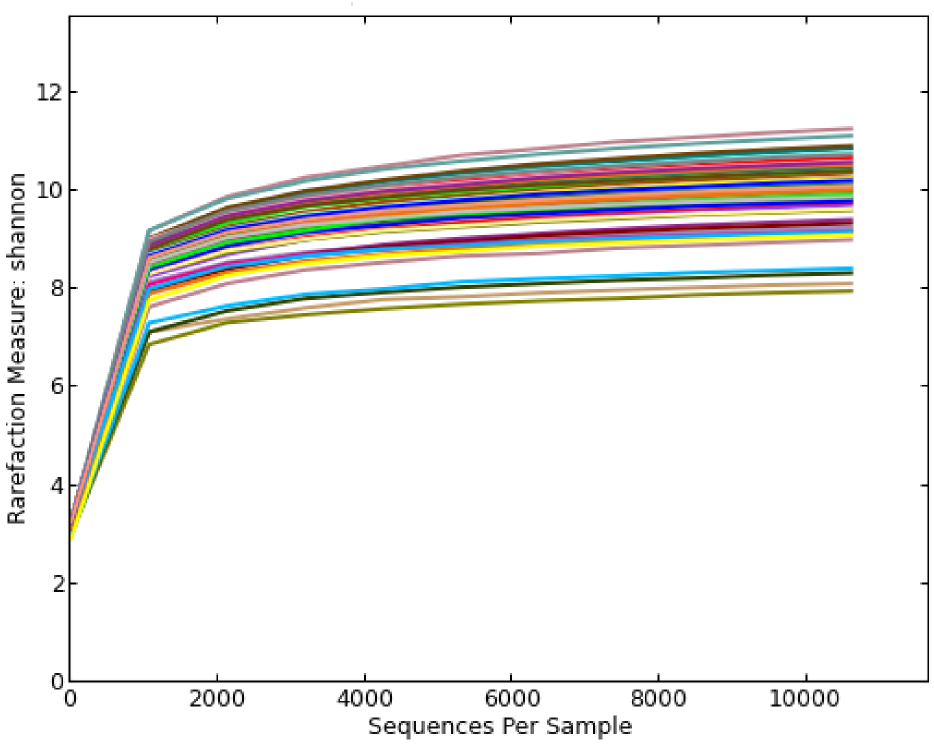

Supplementary Figure S4. Rarefaction curve of Shannon-Weiner index for all samples.

Supplementary Table S1. Correlation matrix for microbial richness and environmental variables as revealed by Spearman Correlation analysis.

	MR	Latitude	Longitude	Elevation	MAT	MAP	PR	PB	Temperature	Moisture	Salinity	TC	TOC	TN	C/N	AN	TP	AP	pH	Clay	Silt	Sand
MR	1.000																					
Latitude	.074	1.000																				
Longitude	-.155	.801**	1.000																			
Elevation	-.064	-.919**	-.799**	1.000																		
MAT	-.065	-.980**	-.828**	.936**	1.000																	
MAP	-.010	-.944**	-.888**	.943**	.980**	1.000																
PR	.341**	.126	-.182	-.104	-.151	-.050	1.000															
PB	.104	.202	.032	-.107	-.202	-.171	.341**	1.000														
Temperature	.045	.652**	.446**	-.710**	-.621**	-.617**	-.063	-.034	1.000													
Moisture	.133	-.183	-.153	.075	.146	.093	.050	.020	-.266*	1.000												
Salinity	.371**	.444**	.132	-.473**	-.437**	-.379**	.230	.010	.394**	.244	1.000											
TC	-.024	-.680**	-.571**	.705**	.639**	.663**	.054	-.056	-.680**	.200	-.350**	1.000										
TOC	.141	.331**	.360**	-.453**	-.346**	-.368**	.031	.069	.252	.146	.286*	-.124	1.000									
TN	-.095	-.538**	-.430**	.584**	.495**	.503**	-.041	-.018	-.587**	.147	-.439**	.883**	-.227	1.000								
C/N	-.136	-.745**	-.585**	.764**	.721**	.732**	.053	-.120	-.731**	.110	-.422**	.771**	-.399**	.574**	1.000							
AN	.252	.622**	.448**	-.588**	-.622**	-.589**	.052	.112	.494**	-.134	.362**	-.323*	.415**	-.245	-.583**	1.000						
TP	-.160	-.460**	-.355**	.479**	.456**	.399**	-.256*	-.117	-.273*	.170	-.390**	.237	-.281*	.327*	.222	-.329**	1.000					
AP	-.011	.308*	.299*	-.403**	-.290*	-.311*	-.180	-.048	.506**	-.196	.240	-.493**	.226	-.430**	-.448**	.219	-.177	1.000				
pH	.280*	.721**	.502**	-.734**	-.709**	-.677**	.121	-.034	.632**	-.205	.534**	-.669**	.116	-.589**	-.697**	.571**	-.514**	.409**	1.000			
Clay	.070	.556**	.447**	-.610**	-.525**	-.547**	-.122	-.067	.588**	-.180	.337**	-.806**	.181	-.756**	-.611**	.348**	-.080	.545**	.684**	1.000		
Silt	.059	.432**	.354**	-.512**	-.409**	-.438**	-.167	-.141	.494**	-.127	.256*	-.616**	.280*	-.544**	-.552**	.339**	.105	.567**	.539**	.903**	1.000	
Sand	-.063	-.443**	-.361**	.521**	.419**	.446**	.161	.143	-.503**	.129	-.267*	.626**	-.272*	.554**	.556**	-.340**	-.094	-.566**	-.553**	-.909**	-.999**	1.000

*: Significant at $P < 0.05$. **: Significant at $P < 0.01$.

MR: microbial richness.
